# The Doctor's New Role

**Published:** 1921-02-19

**Authors:** 


### A MEMORABLE EXHIBITION.

### THE DOCTOR'S NEW ROLE.

The doctor, who used to be regarded as the friend of man, is in danger of being looked upon merely as the servant of industry. At a conference at the Efficiency Exhibition addressed by medical men and health reformers, a resolution was unanimously agreed to calling attention to the necessity of propaganda bearing on dental caries, the prevention and treatment of venereal disease, tuberculosis, the danger of the single sacramental cup, the necessity for a clean bill of health for those wishing to marry, legislation against such evils as the rubber "dummy," and the importance of proper food, good housing, and recreation grounds.

To anyone who has failed to recognise the signs of the times, it is an extraordinary revelation of the variety of the points at which the medical profession touches life and of the demands made upon the profession in the name of efficiency. The appointment of medical men in factories and schools, first as inspectors and then as clinical workers, began the recognition of medicine as an aid to efficiency, but the field is now so wide that it makes the doctor into a modern Altas bearing the weight of empire upon his shoulders. The "white man's burden" is small compared to the medical man's load, for the doctor has to support both the white man and his burden. It is a striking realisation of the fact that the medical profession is by no means a mere agent of pity and succour, but one of the fundamental bases of modern society.

The doctor touches life at all points, and he touches it nowadays as a creative factor, no longer regarded as a regrettable necessity but as an essential accessory in any efficient undertaking where human beings function in any way. The war brought the lesson home to the nation that the doctor was a soldier. Such a resolution as the one quoted marks a realisation that the doctor is also a soldier of industry, that the nurse is contributing to the nation's stocks and its wealth as infallibly as her patient who returns to manage a business or take his place at a bench. This recognition of the profession of medicine as an essential part of efficient industry is a welcome tribute, but as John Wesley said "The world is my parish," the medical man must insist "Life is my domain." It would be fatal to his place in society as well as to the finest work if the medical man should allow his task to be regarded merely as that of keeping workers fit to work. Leisure is life as well as work, and the idea underlying, in this strangely assorted resolution, the reference to recreation ground and proper food is clearly that what the doctor has to do for mankind is to enable it to live the full life. It is not enough to keep a man working his best. His doctor has to keep him playing his best. Too often circumstances suggest that the only concern of the medical man is to get his patient back to work. Common phraseology supports a view too widely if not very definitely held.

The recognition of the importance of the doctor to efficiency should not overcast the importance of the doctor to life. His interest in his patient is not as a temporarily inefficient unit in a workshop or even as a temporarily incapacitated member of a football team. A sound mind in a sound body has always been the doctor's ideal, and he should go beyond it and aim at a happy mind in a healthy body, for though the doctor may be essential to industry, his real task is to be, in a very broad sense, the guardian of mankind.

